# Supplementary figures and images for: A New Species of the Pythonomorph Carentonosaurus from the Cenomanian of Algora (Guadalajara, Central Spain)
Source: Animals (Basel). 2023 Mar 29;13(7):1197. doi: 10.3390/ani13071197 (PMC10093179; doi:10.3390/ani13071197)

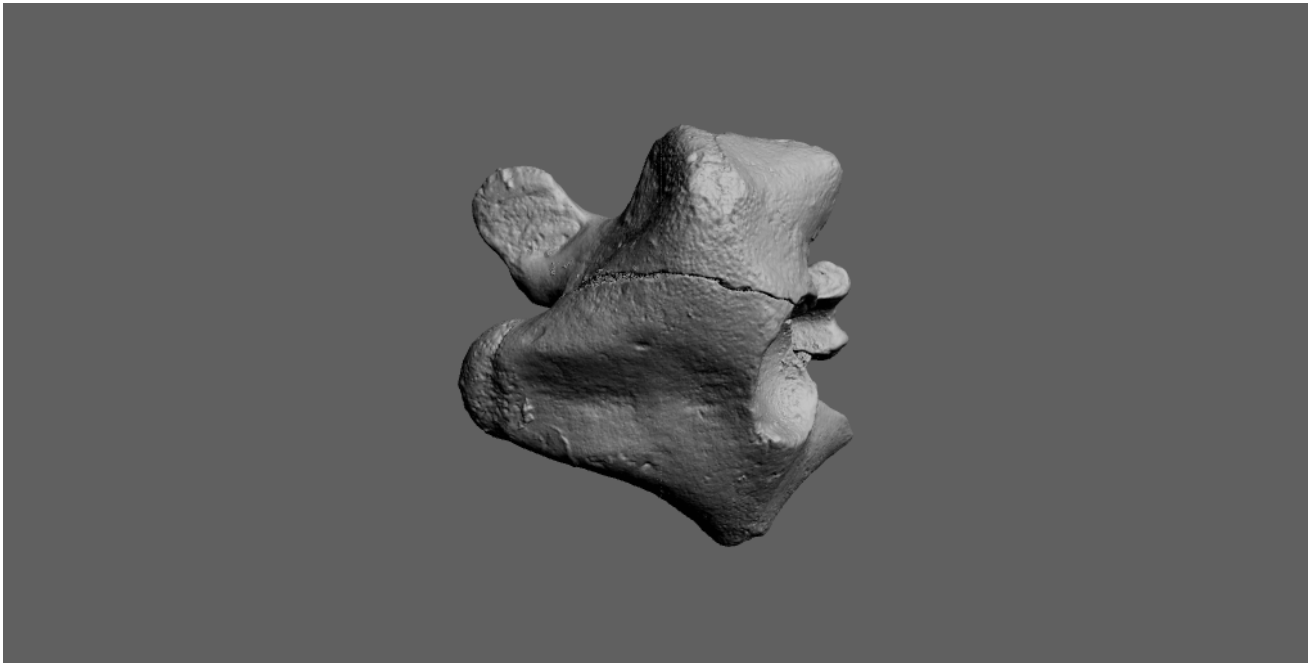

Supplement: Supplementary file 1 [file animals-13-01197-s001.zip › Supplementary File S1.pdf]
